# Childhood fever: Parental paracetamol administration after consulting out-of-hours general practice

**DOI:** 10.1080/13814788.2019.1676415

**Published:** 2019-10-16

**Authors:** Eefje G. P. M. de Bont, Jolijn M. H. A. Bohnen, Rachèl Verhoeven, Geert-Jan Dinant, Jochen W. L. Cals

**Affiliations:** Department of Family Medicine, CAPHRI School for Public Health and Primary Care, Maastricht University, Maastricht, The Netherlands

**Keywords:** Child, infection, general practice, paracetamol

## Abstract

**Background:** Current guidelines emphasise prudent use of paracetamol in febrile children without pain. Little evidence is available on paracetamol administration by parents in general and post-GP-consultations.

**Objectives:** To investigate if and how often parents of febrile children administer paracetamol to their child after consulting a GP during out-of-hours care. To explore if condition (painful or not), socio-economic status and age influenced this behaviour.

**Methods:** This was a pre-planned secondary study, attached to an RCT (*n* = 25,355) that studied the effect of an illness-focused interactive booklet on antibiotic prescriptions in febrile children between three months and 12 years, at 20 GP out-of-hours centres across the Netherlands. Baseline data and ICPC codes were retrieved from the GP out-of-hours centre database. During a telephone survey two weeks after consulting a GP out-of-hours centre, a random sample of parents was asked if and how often they had given their child paracetamol.

**Results:** Parents of 548 children participated. Most parents administrated paracetamol for two weeks after consulting (83.8%). Children received 11 doses on average during follow-up (maximum 72 doses). Paracetamol administration increased with age. Age three to six months received paracetamol in 68% (17/25) of the cases versus 89.6% (121/135) in children aged five to twelve years. Frequency of paracetamol administration was similar for most common infections, regardless of being painful or painless.

**Conclusion:** Most children who consulted out-of-hours general practice for fever and common infections received paracetamol at home during their illness episode, regardless of a painful condition being present. Paracetamol administration increased with age.

KEY MESSAGES

- Current guidelines emphasise a reluctant use of paracetamol in febrile children without pain
- This study shows that at least eight out of ten febrile children - regardless of having a painful condition - receive paracetamol after visiting a GP
- Therefore, GPs could inform parents more explicitly about this issue

## Introduction

Concerns about fever lead to excessive and incorrect use of paracetamol by parents in feverish children [1]. According to several guidelines, the only proven indication to give paracetamol in children with fever is in combination with pain. Previous studies have, therefore, emphasised that fever should be treated independently from the body temperature being driven by the children’s distress [2]. From daily practice, we know that parents frequently give their children paracetamol when they have a fever, even if it this is not necessary or recommended [3,4]. Also, up to 50% of parents might give their child an incorrect dosage of paracetamol, which can be ineffective as well as hepatotoxic [1–4]. However, little actual evidence is available about the administration of paracetamol by parents to febrile children in general and there is no literature on post-GP-consultation paracetamol administration.

In a previously conducted RCT, we studied the effect of an illness-focused interactive booklet on antibiotic prescriptions in febrile children between three months and 12 years old at 20 GP out-of-hours centres across the Netherlands. The booklet also included information on paracetamol use [5].

Since there have been no studies examining paracetamol administration behaviour in children directly after consultations with GPs, our aim with this secondary study – attached to the RCT – was therefore to investigate if and how often parents of febrile children administer paracetamol to their child after consulting a GP during out-of-hours care and if indication (painful condition or not), socio-economic status and age influenced this.

## Methods

### Study design and setting

This study was part of a larger cluster randomised controlled trial (RCT) published and described previously [5,6]. In a multistage process and based on previous qualitative research and existing guidelines, we developed an illness-focused interactive booklet on childhood fever [5]. The primary aim of the original cluster RCT was to study the effect of the illness-focused interactive booklet on antibiotic prescriptions in GP out-of-hours general practice.

The booklet contained – among other things – a weight-banded paracetamol dosage scheme to help parents provide their child with a safe, yet effective dose of antipyretics. For the current pre-planned secondary study, data was collected on paracetamol administration by parents.

From November 2015 to May 2016, parents of children between the age of three months and 12 years old with a fever (at home prior to consulting or during the consultation) were recruited. Twenty large, both rural and urban, GP out-of-hours centres across the Netherlands were recruited.

The trial was approved by the Ethics Committee of Zuyderland-Zuyd (METC Z) in Heerlen, The Netherlands (Ref 14-N-171).

### Data collection

Data on baseline characteristics and International Classification of Primary Care (ICPC) codes were retrieved from the GP out-of-hours centre database. When finalising a consultation, GPs were obliged to choose one ICPC code that was most fitting and specific for the main complaint or diagnosis. ICPC codes that were considered painful conditions were H01.00 Earache, H70.00 Otitis externa, H71.00 Acute otitis media, R21.00 Throat symptoms, R22.00 Tonsillar Symptoms, R74.02 Acute pharyngitis, R76.00, R76.01 Acute tonsillitis, and R77.00 Acute laryngitis.

In addition, data on secondary outcomes was collected among a subsample of parents using telephone surveys during three two-week periods. In these three periods, which were randomly chosen, parents consulting participating GP out-of-hours centres were asked if they would consent with a telephone survey two weeks after the consultation. If parents in this subsample gave written informed consent, a research nurse called them two weeks after the index consultation. During the telephone survey, which lasted 5 min, parents were asked about several outcomes for the primary study (including the child’s recovery, consultations with their GP, complications). For the current study specifically, they were asked how often they had given their child paracetamol (yes/no and frequency) in the two weeks following the initial fever-related consultation.

### Outcome

The outcome variables of this study were the percentage of paracetamol administration (yes/no) by parents of a febrile child and the quantity of paracetamol given during two weeks after the initial fever-related consultation at the out-of-hours GP centre.

Although analysing the effect of the booklet on paracetamol administration was not our goal, we cannot rule out any influence of the booklet. Therefore, we choose to show results for the three subgroups in the primary study, being: 1. usual care group (no booklet); 2. GPs having access to the booklet; 3. GPs who had access and actually used the booklet. Between-group differences were investigated.

### Data analysis

Data analyses were performed with IBM SPSS Statistics 21.0 using descriptive statistics and frequency tables to summarise the data. Secondly, statistical analysis was done by fitting two level (GP out-of-hours centre and patient) random intercept logistic regression models to correct for the cluster effect with MLwiN for the total sample with paracetamol (yes/no) as a dependent variable and age, SES, indication and painful condition specifically as independent variables.

## Results

### Population characteristics

In the primary study, a total of 29,364 fever-related consultations took place among 25,355 individual children. Of these children, 553 parents provided written informed consent to participate in the telephone survey (*n* = 250 control group, *n* = 303 access to booklet group, of which use of booklet group *n* = 109) [5,6]. Distribution of gender, age and SES is presented in Table 1 and were similar between the three groups. The sample recruited for the telephone interviews were comparable to the complete sample of children recruited in the large cluster randomised controlled trial (Table 1). The most frequently used ICPC codes were A03.00 (fever), H71.00 (acute otitis media) and R74.00 (acute respiratory tract infection), which were also comparable distributed between groups.

**Table 1.** Baseline characteristics between groups.

	Access to booklet (*n* = 303)	Use of booklet (*n* = 109)	Control (*n* = 250)	Total (*n* = 553)	Complete RCT (*n* = 25,355)
Mean age (years, SD)	3.12 (2.7)	2.78 (2.6)	2.78 (2.6)	2.97 (2.6)	3.2 (2.7)
Male sex N (%)	153 (50.5)	36 (50.0)	143 (57.2)	296 (53.5)	13,413 (52.9)
ICPC code N (%)					
A03.00 (fever)	62 (20.5)	28 (25.7)	51 (20.4)	113 (20.4)	4,654 (18.4)
H71.00 (OMA)	44 (14.5)	15 (13.8)	41 (16.4)	85 (15.4)	5,010 (19.8)
R74.00 (RTI)	69 (22.8)	29 (26.6)	56 (22.4)	125 (22.6)	3,476 (13.8)
Other	126 (41.6)	35 (32.1)	101 (40.4)	227 (41.0)	–
SES N (%)*					
Low	40 (13.2)	18 (16.5)	37 (14.8)	41 (12.7)	4,087 (16.2)
Middle	217 (71.6)	76 (69.7)	189 (75.6)	243 (75.2)	17,514 (69.5)
High	43 (14.2)	14 (12.8)	24 (9.6)	38 (11.8)	3,582 (14.2)

*Three cases were missing for SES in the access to booklet group and one case was missing in the use of booklet group.

### Paracetamol administration

**Figure 1.**
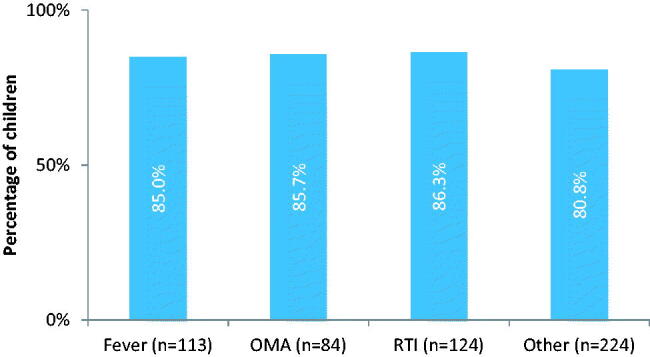
Children given paracetamol at least once for different ICPC codes during 2 weeks follow-up, no significant difference between groups (*p* Value 0.1 for highest percentage in comparison to lowest).

**Figure 2.**
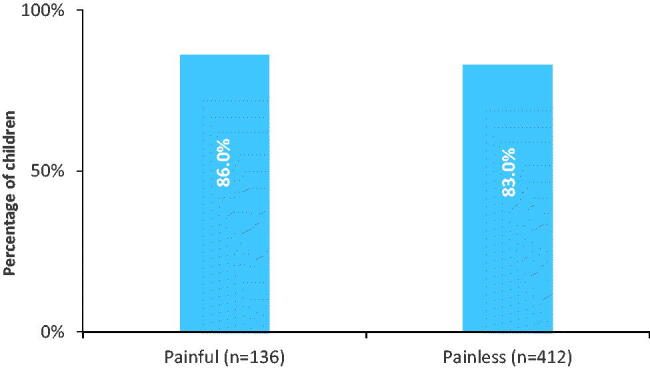
Children given paracetamol for painless versus painful* conditions during 2 weeks follow-up, no significant difference between groups (*p* Value 0.41)*; painful conditions: see Methods section.

We had data on paracetamol administration for 548/553 eligible children. In total, 83.8% (459/548) of the parents gave their child paracetamol at least once during two weeks of follow up. The frequency varied between 1 to 72 times with a mean of 10.7 times. Paracetamol was administered almost equivalent for different ICPC codes, regardless of being a painful versus painless condition (Figures 1 and 2). When examining painful versus non-painful ICPC codes in more detail, 136 had a painful condition, of which 117 received paracetamol (86%, Figure 2). The remaining children were not classified as having a painful condition and received paracetamol in 83% of the cases. They were most commonly classified as having other viral infections.

**Figure 3.**
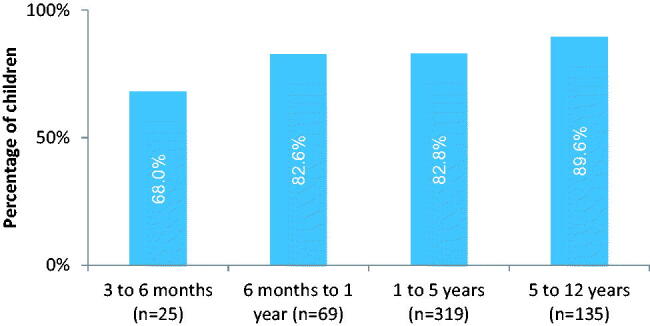
Children given paracetamol at least once for different age categories during 2 weeks follow-up, significant increase with age (*p* Value 0.04).

Paracetamol was administered more often to older children with a percentage of 89.6% for children 5–12 years old (121/135, Figure 3), regardless of the randomised groups. No significant influence for SES or antibiotic prescription was observed on paracetamol administration (yes/no).

## Discussion

### Main findings

This study shows that 84% of parents gave their feverish child paracetamol at least once within two weeks after consulting a GP during out-of-hours care, regardless of whether their child suffered from a painful (86%) or painless (83%) condition. On average, children received 11 doses in two weeks, with peaks of 50 and 72 doses. Paracetamol administration increased with age: from 68% in children aged 3–6 months to 90% in children aged five to twelve years.

### Comparison to existing literature

The findings of this study in terms of frequency of paracetamol administration to children are in line with findings of previous studies in different settings. Most of these previously performed studies show a high percentage of paracetamol administration by parents, ranging from 60–97% [7,8].

To our knowledge, this is the first study also studying the relationship between potential painful versus painless diagnosis and paracetamol administration after GP consultations. We found no difference between painful versus painless diagnosis and parental paracetamol administration after consultations. This fits the conclusion of a recent review that states that up to 60% of parents reported using drugs to control the level of temperature rather than to relieve discomfort [9].

These new findings are important because we know that many parents medicate their febrile child extensively with paracetamol because of concerns and anxiety for severe complications, like febrile seizures [1,8]. In addition, there are studies showing that parents believe general well-being of their child is improved by paracetamol, allowing them to become more alert, had an increased appetite and fell asleep more easily [7]. However, only a few studies investigated the effect of paracetamol on the well-being of feverish children and those have shown no significant effect [10]. Paracetamol might, therefore, also be a way of actively doing something and coping with a sick child in a busy daily life [7].

### Strengths and limitations

The strength of this study is that it is the first study investigating paracetamol administration by parents of feverish children right after visiting the GP out-of-hours centre. The most important limitation of our study is the fact that we depended on physicians’ ICPC coding for reasons of encountering. Although this is the first study distinguishing painful conditions from painless conditions, we did not have in-depth information why parents did or did not provide their child with paracetamol. In addition, there could have been recall bias since we did not register paracetamol administration at the moment it was given. We also do not know how many parents did not consent to participate in the telephone survey and what their reasons were. Likely, parents of generally more unwell children did not have time to fill in the informed consent in the waiting room of the out-of-hours centre.

### Implications for research and practice

This study, therefore, calls for a more in-depth research on reasons why parents give their child paracetamol in childhood fever and how this relates to pain or discomfort.

It is up to physicians to prevent medicalisation of fever as a disease itself. Especially since research shows that the amount of paracetamol administration not only depends on the height of the temperature and instructions on the medication box but also importantly on physicians’ instructions [11]. In theory, by de-medicalising fever as an entity by itself, parental consultation frequency could also be influenced since fever not responding to paracetamol is a frequently mentioned reason for health care-seeking behaviour.

## Conclusion

This study shows that many parents administer paracetamol to their feverish child regardless of whether it suffers from a painful condition or not. This suggests that parents may not always give paracetamol for an appropriate reason. Apart from explaining age-dependent dosing strategies, GPs could inform parents about the correct indication for paracetamol in febrile children, being pain.
